# Awareness, Training, and Perceived Needs of Gynecologists in Interpreting Basic Imaging Studies: A Cross-Sectional Survey in Saudi Arabia

**DOI:** 10.7759/cureus.73748

**Published:** 2024-11-15

**Authors:** Eman Altom, Aminah Fouad, Doaa Bilal, Njood Alsudairy

**Affiliations:** 1 General Practice, Maternity and Children Hospital, Najran, SAU; 2 Radiology, Second Health Cluster, Jeddah, SAU

**Keywords:** clinical decision-making, ct, gynecologists, imaging interpretation, medical education, mri, radiology, saudi arabia, training needs, ultrasound

## Abstract

Background: Gynecologists often rely on imaging studies such as ultrasound, CT, and MRI for clinical decision-making, yet limited training in interpreting these studies may affect their confidence and ability to make timely diagnoses. This study aimed to assess the awareness, training and perceived needs of gynecologists in Saudi Arabia regarding the interpretation of basic imaging studies.

Methods: A cross-sectional survey was conducted among 200 gynecologists practicing in Saudi Arabia. Participants were recruited using convenience sampling, and data were collected through an online questionnaire that assessed demographics, imaging knowledge, training history, perceived barriers, and interest in further education. Descriptive statistics were used to analyze the data.

Results: The majority of respondents (44%) reported interpreting imaging studies occasionally, with ultrasound being the most commonly interpreted modality (63%). However, only 29% had received formal training in imaging studies, and 74% felt their training was insufficient. Most respondents (82%) expressed interest in additional training, particularly in ultrasound interpretation and emergency imaging. Key barriers to effective imaging interpretation included lack of training (43%) and reliance on radiology reports (24%). Nearly half (45%) of participants noted that delays in radiology reports affected their clinical decision-making.

Conclusions: This study reveals significant gaps in imaging interpretation training among gynecologists in Saudi Arabia, with a high demand for further education in basic imaging modalities. Addressing these gaps through structured training programs could improve gynecologists’ confidence and clinical decision-making, ultimately leading to better patient care outcomes.

## Introduction

Gynecologists frequently encounter clinical scenarios where basic imaging studies, such as ultrasound, CT, and MRI, play a critical role in diagnosis, management, and treatment planning. Ultrasound, in particular, is commonly used in gynecology for evaluating pregnancy, pelvic masses, and other gynecologic conditions due to its accessibility, affordability, and safety profile [1,2]. Although gynecologists often rely on radiology reports for interpretation, the ability to independently interpret basic imaging findings could enable more timely and effective patient care, particularly in settings where radiologists are not immediately available [3,4].

In Saudi Arabia, as in many parts of the world, there has been increasing recognition of the importance of cross-disciplinary skills among healthcare providers. Several studies have highlighted that enhanced imaging interpretation skills among non-radiologists could improve clinical decision-making and patient outcomes. However, research examining gynecologists’ specific imaging interpretation skills, confidence levels, and training experiences remains limited. Previous surveys have shown variability in the extent of imaging training received during residency, often with limited structured instruction beyond ultrasound. This gap can lead to decreased confidence in interpreting imaging findings, potential delays in diagnosis, and an increased dependence on radiologists for basic assessments [5-7].

Understanding the current level of awareness, training, and confidence among gynecologists in interpreting imaging studies is essential for informing targeted educational interventions. Given the evolving healthcare needs in Saudi Arabia and the global emphasis on integrative clinical skills, this study aims to assess the current imaging interpretation competencies, barriers, and training needs of gynecologists practicing in Saudi Arabia. By identifying specific gaps and priorities, the findings from this study may inform future educational programs, ultimately enhancing patient care and clinical efficiency in gynecologic practice.

## Materials and methods

Study design and setting

This cross-sectional survey-based study was conducted in Saudi Arabia to assess the awareness, training, and perceived needs of gynecologists in interpreting basic imaging studies. The Ethics Committee, Ministry of Health, Saudi Arabia issued approval 2024-9955. Data were collected between April and June 2024. The study targeted practicing gynecologists in both private and public healthcare settings, including academic hospitals, private practices, and rural health centers. Informed consent was collected from each participant prior to data collection, reinforcing the commitment to ethical research practices.

Sample size and participant recruitment

A target sample size of 200 gynecologists was set based on the estimated regional distribution and accessibility of gynecologists in Saudi Arabia. Participants were recruited using convenience sampling methods through professional networks, healthcare institutions, and professional associations. The survey link was disseminated via email and social media platforms commonly used by healthcare professionals in Saudi Arabia. Participation was voluntary, and informed consent was obtained from all respondents before the survey.

Survey instrument

The survey questionnaire was developed based on the study objectives and included six sections: demographics, current awareness and knowledge of imaging, prior training in imaging studies, perceived barriers to imaging interpretation, impact on patient care, and future training needs. All questions were closed-ended, using multiple-choice and Likert-scale response options, to ensure ease of completion and consistency in responses. The questionnaire was initially piloted with a small group of gynecologists (n=10) to ensure clarity, relevance, and cultural appropriateness. Minor adjustments were made based on feedback from the pilot group.

Data collection and analysis

Data collection was conducted electronically using Google Forms. The survey link remained open for a specified duration of four weeks to maximize response rates. Reminder emails were sent weekly to encourage participation. Respondents were assured of the confidentiality and anonymity of their responses. Descriptive statistics were used to summarize the responses. Categorical variables, such as demographic characteristics and response frequencies, were presented as counts and percentages.

## Results

Demographics and professional background

A total of 200 gynecologists participated in the survey. The majority practiced in academic/teaching hospitals (72, 36%) or private practices (68, 34%), with fewer working in government/public hospitals (46, 23%) and rural health centers (14, 7%) (Table 1). Most respondents had between 5-10 years of experience (64, 32%) or 11-20 years of experience (58, 29%), while 45 (22.5%) had <5 years and 33 (16.5%) had >20 years of experience. Additionally, only 54 (27%) reported having subspecialty training, while 146 (73%) did not (Table 1).

**Table 1:** Demographics and professional background of respondents (N=200). This table summarizes the demographic and professional characteristics of survey respondents, including their place of practice, years of experience, and subspecialty training.

Characteristic		n (%)
Primary place of practice	Academic/teaching hospital	72 (36%)
	Private practice	68 (34%)
	Government/public hospital	46 (23%)
	Rural health center	14 (7%)
Years of practice	<5 years	45 (22.5%)
	5–10 years	64 (32%)
	11–20 years	58 (29%)
	>20 years	33 (16.5%)
Additional subspecialty training	Yes	54 (27%)
	No	146 (73%)

Awareness and current knowledge of imaging studies

Nearly half of the respondents reported occasionally interpreting imaging studies in clinical practice (88, 44%), while 58 (29%) rarely did so, and only 32 (16%) frequently interpreted imaging. Ultrasound was the most familiar imaging modality (126, 63%), followed by CT (34, 17%) and MRI (24, 12%). Notably, 16 (8%) reported no familiarity with any imaging modality. In terms of confidence, 88 (44%) were “somewhat confident” in interpreting imaging, while 52 (26%) were “confident,” and 22 (11%) were “very confident.” However, 38 (19%) admitted to having no confidence in this skill (Table 2).

**Table 2:** Awareness and current knowledge of imaging studies among gynecologists (N=200). This table outlines the respondents’ familiarity and confidence with interpreting various imaging modalities used in gynecology.

Question	Response options	n (%)
Frequency of interpreting imaging studies	Never	22 (11%)
	Rarely (<10% of cases)	58 (29%)
	Occasionally (10–30% of cases)	88 (44%)
	Frequently (>30% of cases)	32 (16%)
Most familiar imaging modality	Ultrasound	126 (63%)
	CT scan	34 (17%)
	MRI	24 (12%)
	None	16 (8%)
Confidence in interpreting imaging	Not confident	38 (19%)
	Somewhat confident	88 (44%)
	Confident	52 (26%)
	Very confident	22 (11%)

Training and education in imaging studies

Formal training in imaging studies was received by 58 (29%) of respondents during residency and 42 (21%) through post-residency workshops or courses, while 100 (50%) reported no formal training. Among those trained, only 52 (26%) felt their training was sufficient, whereas 148 (74%) did not. Encouragingly, 164 (82%) expressed interest in additional training, with the preferred formats being in-person workshops (82, 41%) and online courses (60, 30%) (Table 3).

**Table 3:** Training and education in imaging studies (N=200).

Question	Response options	n (%)
Formal training in imaging studies	Yes (during residency)	58 (29%)
	Yes (post-residency workshops or courses)	42 (21%)
	No	100 (50%)
Was training sufficient?	Yes	52 (26%)
	No	148 (74%)
Interest in additional training	Yes	164 (82%)
	No	36 (18%)
Preferred training format	In-person workshops	82 (41%)
	Online courses	60 (30%)
	Mentorship/shadowing	42 (21%)
	Reading materials/self-paced modules	16 (8%)

Perceived barriers to interpreting imaging studies

The most common barrier to interpreting imaging studies was a lack of training (86, 43%), followed by reliance on radiology reports (48, 24%), time constraints (42, 21%), and limited access to imaging (24, 12%) (Table 4).

**Table 4:** Perceived barriers to interpreting imaging studies (N=200). This table identifies the primary challenges faced by gynecologists in interpreting imaging studies effectively.

Primary barrier to interpreting imaging studies	n (%)
Lack of training	86 (43%)
Time constraints	42 (21%)
Reliance on radiology reports	48 (24%)
Limited access to imaging studies	24 (12%)

Impact of imaging interpretation on patient care

Most respondents believed that enhanced imaging interpretation skills could improve patient outcomes, with 92 (46%) strongly agreeing and 74 (37%) agreeing (Table 5). Conversely, only eight (4%) disagreed and six (3%) strongly disagreed. Regarding the impact of radiology reporting delays on clinical decision-making, 90 (45%) reported being affected “sometimes,” 72 (36%) “often,” and 18 (9%) “always.”

**Table 5:** Perceptions of the impact of imaging skills on patient care (N=200). This table captures respondents’ views on the significance of imaging interpretation skills in improving patient outcomes and the effect of delays in radiology reporting.

Question	Response options	n (%)
Enhanced skills improve outcomes	Strongly agree	92 (46%)
	Agree	74 (37%)
	Neutral	20 (10%)
	Disagree	8 (4%)
	Strongly disagree	6 (3%)
Delays in radiology affect decision-making	Always	18 (9%)
	Often	72 (36%)
	Sometimes	90 (45%)
	Rarely	20 (10%)

Future directions for imaging training

When asked about priorities for training, the majority identified the basics of ultrasound interpretation (124, 62%) and identifying common pathologies on imaging (112, 56%) as the most important areas. Other areas of interest included emergency imaging such as ectopic pregnancy diagnosis (102, 51%), cross-sectional imaging basics (88, 44%), and radiation safety (66, 33%). Nearly all respondents (180, 90%) were willing to participate in structured training programs to enhance their imaging interpretation skills (Table 6).

**Table 6:** Future directions for imaging training among gynecologists (N=200). This table explores respondents’ priorities for imaging training and willingness to participate in structured educational programs.

Question	Response options	n (%)
Prioritized areas for imaging training	Basics of ultrasound interpretation	124 (62%)
	Identifying common pathologies	112 (56%)
	Emergency imaging (e.g., ectopic pregnancy)	102 (51%)
	Cross-sectional imaging (CT/MRI) basics	88 (44%)
	Radiation safety	66 (33%)
Willingness to participate in training	Yes	180 (90%)
	No	20 (10%)

## Discussion

This study highlights the current status of imaging interpretation skills, training experiences, and perceived needs among gynecologists in Saudi Arabia. Our findings reveal substantial gaps in formal imaging training and confidence levels, with a strong interest among respondents for additional educational opportunities. The majority of gynecologists reported occasional to rare involvement in interpreting imaging studies, with ultrasound being the most familiar modality. However, only a minority expressed high confidence in their interpretation skills, underscoring a need for more structured training.

Our results show that only half of the respondents had received any formal training in imaging interpretation, primarily during residency, and nearly three-quarters felt this training was insufficient. This finding aligns with similar international studies, which suggest that imaging training for non-radiologists is often limited in both scope and depth. Given that ultrasound is widely used in gynecology, many respondents indicated familiarity with this modality, yet fewer reported confidence in interpreting CT or MRI. These findings suggest a need for broader and more specialized imaging education for gynecologists, particularly given the increasing reliance on cross-sectional imaging in the management of gynecologic conditions [6-9].

Lack of training emerged as the primary barrier, with respondents also citing reliance on radiology reports, time constraints, and limited imaging access. The reliance on radiologists for basic interpretations can delay decision-making and complicate care, especially in settings where radiologists are not readily available. Addressing this gap through targeted training programs could potentially enhance clinical efficiency and improve patient care outcomes by reducing these dependencies [10,11].

Most respondents agreed that enhanced imaging interpretation skills could improve patient outcomes, a finding that aligns with the increasing emphasis on integrated care and cross-disciplinary competencies in healthcare. Nearly half of the respondents noted that delays in receiving radiology reports often affect their clinical decision-making, which highlights the potential benefit of empowering gynecologists with the skills to independently interpret basic imaging studies. In particular, familiarity with emergency imaging findings, such as ectopic pregnancies, and other critical pathologies could support faster and more effective treatment in urgent scenarios [12-14].

Encouragingly, the majority of respondents expressed a desire for additional training in imaging interpretation, with preferences for in-person workshops and online courses. This interest reflects a broader trend in healthcare, where practitioners seek accessible, flexible, and practical educational programs. The prioritized areas for training, such as ultrasound basics, emergency imaging, and identifying common pathologies, align with the core needs of gynecologic practice and could be effectively addressed through competency-based training modules tailored to the unique needs of gynecologists [6,8].

Limitations and future directions

This study has several limitations, including its reliance on self-reported data and the potential for selection bias, as respondents may have been more likely to participate if they had a particular interest in imaging. Additionally, the survey was conducted in Saudi Arabia, and while findings may reflect challenges and needs relevant to the regional healthcare context, further research across diverse healthcare settings could provide a broader perspective on gynecologists’ imaging training needs globally.

## Conclusions

In conclusion, this study highlights significant gaps in the training, awareness, and confidence of gynecologists in Saudi Arabia regarding the interpretation of basic imaging studies. Despite the widespread use of ultrasound in gynecologic practice, many gynecologists report limited formal training and lack confidence in interpreting more advanced imaging modalities like CT and MRI. There is a strong interest among practitioners for additional training, particularly in ultrasound, emergency imaging, and cross-sectional imaging, to improve clinical decision-making and patient care. Addressing these educational needs through structured, accessible training programs could enhance the clinical competence of gynecologists, reduce reliance on radiology reports, and ultimately contribute to better patient outcomes in gynecology.

## References

[1] Abuhamad A, Minton KK, Benson CB (2018). Obstetric and gynecologic ultrasound curriculum and competency assessment in residency training programs: consensus report. J Ultrasound Med.

[2] Recker F, Gembruch U, Strizek B (2024). Clinical ultrasound applications in obstetrics and gynecology in the year 2024. J Clin Med.

[3] Lee W, Hodges AN, Williams S, Vettraino IM, McNie B (2004). Fetal ultrasound training for obstetrics and gynecology residents. Obstet Gynecol.

[4] Yerra AK, Jogi S, Emmadisetty S, Animalla V, D'souza A (2023). Simulation-based training on basic obstetrics and gynecology ultrasound skills during COVID pandemic. J Obstet Gynaecol India.

[5] Weimer J, Recker F, Hasenburg A (2024). Development and evaluation of a "simulator-based" ultrasound training program for university teaching in obstetrics and gynecology-the prospective GynSim study. Front Med (Lausanne).

[6] Lei T, Zheng Q, Feng J (2024). Enhancing trainee performance in obstetric ultrasound through an artificial intelligence system: randomized controlled trial. Ultrasound Obstet Gynecol.

[7] McCurdy RJ, High B, Schnatz PF, Baxter J, Jiang X (2019). Transvaginal ultrasound training for the obstetrics and gynecology resident: a multisite randomized controlled trial of educational DVD. J Clin Ultrasound.

[8] Leonardi M, Ong J, Espada M, Stamatopoulos N, Georgousopoulou E, Hudelist G, Condous G (2020). One-size-fits-all approach does not work for gynecology trainees learning endometriosis ultrasound skills. J Ultrasound Med.

[9] Pinto A, Giurazza F, Califano T, Rea G, Valente T, Niola R, Caranci F (2021). Interventional radiology in gynecology and obstetric practice: safety issues. Semin Ultrasound CT MR.

[10] Recker F, Weber E, Strizek B, Gembruch U, Westerway SC, Dietrich CF (2021). Point-of-care ultrasound in obstetrics and gynecology. Arch Gynecol Obstet.

[11] Jost E, Kosian P, Jimenez Cruz J, Albarqouni S, Gembruch U, Strizek B, Recker F (2023). Evolving the era of 5D Ultrasound? A systematic literature review on the applications for artificial intelligence ultrasound imaging in obstetrics and gynecology. J Clin Med.

[12] Drukker L, Noble JA, Papageorghiou AT (2020). Introduction to artificial intelligence in ultrasound imaging in obstetrics and gynecology. Ultrasound Obstet Gynecol.

[13] Morris JM, Schneider E, Phinehas R (2022). Transvaginal ultrasonography numbers reported by graduating residents in obstetrics and gynecology training programs. Obstet Gynecol.

[14] Papp Z, Fekete T (2003). The evolving role of ultrasound in obstetrics/gynecology practice. Int J Gynaecol Obstet.

